# An Antarctic molluscan biomineralisation tool-kit

**DOI:** 10.1038/srep36978

**Published:** 2016-11-11

**Authors:** Victoria A. Sleight, Benjamin Marie, Daniel J. Jackson, Elisabeth A. Dyrynda, Arul Marie, Melody S. Clark

**Affiliations:** 1 British Antarctic Survey, Natural Environment Research Council, High Cross, Madingley Road, Cambridge, CB3 0ET, UK; 2 Centre for Marine Biodiversity & Biotechnology, Institute of Life & Earth Sciences, Heriot-Watt University, Edinburgh, EH14 4AS, UK; 3 UMR 7245 MNHN/CNRS Molécules de Communication et Adaptation des Micro-organismes, Sorbonne Universités, Muséum National d’Histoire Naturelle, CP 39, 12 Rue Buffon, 75005 Paris, France; 4 Department of Geobiology, Goldschmidtstr.3, Georg-August University of Göttingen, 37077 Göttingen, Germany

## Abstract

The Antarctic clam *Laternula elliptica* lives almost permanently below 0 °C and therefore is a valuable and tractable model to study the mechanisms of biomineralisation in cold water. The present study employed a multidisciplinary approach using histology, immunohistochemistry, electron microscopy, proteomics and gene expression to investigate this process. Thirty seven proteins were identified via proteomic extraction of the nacreous shell layer, including two not previously found in nacre; a novel T-rich Mucin-like protein and a Zinc-dependent metalloprotease. *In situ* hybridisation of seven candidate biomineralisation genes revealed discrete spatial expression patterns within the mantle tissue, hinting at modular organisation, which is also observed in the mantle tissues of other molluscs. All seven of these biomineralisation candidates displayed evidence of multifunctionality and strong association with vesicles, which are potentially involved in shell secretion in this species.

Biominerals are ever-present in the biological world. Across geological time and biological diversity, they are a significant feature of life as we know it^1^. Molluscs owe much of their evolutionary success to their ability to biomineralise and their characteristic shells^2^. With over 85,000 extant species^3^,^4^, molluscs are essential in worldwide ecosystem functions as well as being a source of protein for the growing human population via wild harvest and farming. Aquaculture is the fastest growing food production industry in the world^5^ with mollusc aquaculture making-up approximately 22% (by volume) of global production. In addition, the physical features of the molluscan shell, compared to other composite materials, makes it an attractive low-energy, high-strength, material source, with the advantage that it is formed from a vastly abundant precursor (soluble calcium carbonate). In contrast, there is concern for calcified biological structures in the marine environment due to ocean acidification^6^, and environmental scientists are dedicating much effort to predict the fate of marine calcifiers under future ocean acidification scenarios. There is therefore a growing requirement, from diverse stakeholders, to holistically understand the process of molluscan biomineralisation^7^,^8^,^9^.

The Antarctic clam *Laternula elliptica* is a large and experimentally tractable infaunal mollusc. Its shell has been previously characterised and is composed of 3 structurally distinct layers: (i) the outer periostracum (relatively thick and formed of two layers - approximately 10 μm); (ii) an aragonitic outer layer of granular prisms; and (iii) an aragonitic layer of sheet nacre^10^. *L. elliptica* is a “keystone” species in the Southern Ocean benthic ecosystem and is vulnerable to the multiple effects of climate change. Risks include acidification, warming and the potential for a greater frequency of shell damage by scouring^11^,^12^,^13^ (due to a warming-induced increase of icebergs). *L. elliptica* inhabits cold water (circa 0 °C) and understanding how shells are built in subzero temperatures could provide a theoretical groundwork for both understanding mechanistic responses to climate change, and also the production of innovative new low-temperature biomaterials.

Our previous research has focused on transcriptomic techniques to identify potential biomeralisation gene candidates^14^, which were additionally investigated using shell damage-repair experiments^12^. The aim of the present study therefore, is to further the biological understanding of our previously published sequence data. More specifically the aims of this work are: 1.) To provide a detailed characterisation of the *L. elliptica* mantle anatomy and ultrastructure. 2.) Identify proteins in the *L. elliptica* nacreous shell layer using proteomics and 3.) To increase the understanding of the function of candidate biomineralisation proteins by localising their gene expression at the tissue, cellular and subcellular level.

## Methods

### Animals

*L. elliptica* specimens were collected by SCUBA divers from Hangar Cove near Rothera Research Station, Antarctic Peninsula (67° 34′ 07″ S, 68° 07′ 30″ W) at depths of 10–15 m. Animals were immediately returned to the aquarium where they were maintained in a flow-through system with temperature of 0.6 ± 0.3 °C, under a 12 h:12 h simulated light:dark cycle. Animals were transferred to the British Antarctic Survey aquarium facilities (in the UK) where they were habituated to aquarium conditions for at least four weeks (closed recirculating system at water temperature and salinity of 0 ± 0.5 °C and 35–38 psu respectively, a 12 h:12 h light:dark regime, and fed a mircoalgal mix of *Isochrysis* and *Nannochloropsis* culture weekly) prior to any sampling.

### Mantle anatomy characterisation

Adult *L. elliptica* mantle tissues (n = 5) were fixed in freshly prepared Davidson fixative (22% formalin, 33% ethyl alcohol, 12% glacial acetic acid and 33% sterile sea water), embedded in paraffin wax and sectioned at 8 μm. Tissue sections were rehydrated through a graded ethanol series and stained with haematoxylin and eosin (H&E) as per ref. 15. H&E stained tissue sections were imaged using light microscopy (LM) and used to characterise mantle histology.

For transmission electron microscopy (TEM), adult mantle tissues (n = 3) were fixed by immersion in 2% formaldehyde (made from paraformaldehyde) and 2% vacuum distilled glutaraldehyde, containing 2 mlL^−1^ CaCl_2_, in 0.05 M sodium cacodylate buffer at 4 °C and pH 7.4. The fixative was made isotonic with sea water by the addition of sucrose. Tissues were then removed, sliced to 2–4 mm in one dimension and fixed for an additional 4–6 h at 4 °C. Tissues were then rinsed for 5 × 3 min in cold cacodylate buffer containing 2 mM calcium chloride and incubated in this solution with 1% osmium ferricyanide for 18 h, at 4 °C and rinsed 5 times in deionised water (DIW). This was followed by 30 min in 1% thiocarbohydrazide at room temperature (RT) followed by 5 more rinses in DIW. They were then incubated in 1% uranyl acetate in 0.05 maleate buffer at pH 5.5 and at 4 °C overnight and rinsed with DIW at RT 5 × 3 min, with subsequent dehydration through twice each of 50%, 70%, 90%, and 100% ethanol, followed by twice each in dry ethanol, dry acetone and dry acetonitrile. Samples were then infiltrated with Quetol 651 epoxy resin over a period of 5 d. The resin was cured for 48 h at 65 °C. Thin sections, prepared with a Leica Ultracut S mounted on 200 mesh copper grids, were viewed with a Tecnia G2 operated at 200 kV.

### Proteomic analysis of nacre shell proteins

#### Shell preparation and protein extraction

Superficial organic contaminants and the periostracum were removed by incubating intact adult shells (n = 6) in sodium hypochlorite (5%, vol/vol) for 24–48 h followed by rinsing with water. The external prismatic layer was mechanically removed and the nacre was broken into 1-mm large fragments before being ground to fine powder (>200 μm) and decalcified in acetic acid overnight (10%; 4 °C). The acid-insoluble matrix (AIM) was collected by centrifugation (15,000 g; 10 min; 4 °C) and rinsed six times with MilliQ water by a series of resuspension-centrifugation steps before being freeze-dried and weighed.

Digestions of insoluble nacre matrix samples were performed in a solution of 100 μL of 10 mM dithiothreitol (Sigma-Aldrich, France) in 500 mM triethylammonium bicarbonate (pH 8.0; 30 min; 57 °C). Iodoacetamine (15 μL; 50 mM, final concentration) was added and alkylation was performed (30 min; RT in the dark). Digestion was performed by adding 10 μg of trypsin (T6567, proteomics grade, Sigma). Samples were incubated overnight (37 °C), centrifuged (30 min; 14,000 g) and injected (5 μL of acidified supernatants) into the tandem mass spectrometer with an electrospray source coupled to a liquid chromatography system (LC-ESI-MS/MS).

#### High performance liquid chromatography (HPLC)

HPLC of the tryptic peptides was performed on a C18 micro-column at a flow rate of 50 μL min^−1^ with a linear gradient (10 to 80% in 60 min) of acetonitrile and 0.1% formic acid. Fractionated peptides were analysed in triplicate with an electrospray ionisation quadripole time-of-flight (ESI-QqTOF) hybrid mass spectrometer (pulsar i, Applied Biosystems) using information dependent acquisition (IDA), which allows switching between MS and MS/MS experiments. Data were acquired and analysed with Analyst QS software (Version 1.1). After 1 s acquisition of the MS spectrum, the two most intense multiple charged precursor ions (+2 to +4) could be selected for 2 s-MS/MS spectral acquisitions. The mass-to-charge ratios of the precursor ions selected were excluded for 60 s to avoid re-analysis. The minimum threshold intensity of the ion was set to 10 counts. The ion-spray potential and declustering potential were 5200 V and 50 V, respectively. The collision energy for the gas phase fragmentation of the precursor ions was determined automatically by the IDA based on their mass-to-charge ratio (m/z) values.

#### Nucleotide and amino acid sequence identity

MS/MS data were pooled (from triplicates) and used for database searches using an in house version of Mascot (Matrix Science, London, UK; version 2.1) and PEAKS (Bioinformatics solutions Inc., Waterloo, Canada; version 7.0) search engines against the previously published *L. elliptica* transcriptome^14^. LC-MS/MS data was searched using carbamido-methylation as a fixed modification and methionine oxidation as a variable modification. The peptide MS tolerance was set to 0.5 Da and the MS/MS tolerance was set to 0.5 Da.

Protein sequence characterisation was carried out using BLAST sequence similarity searches against the UniProtKB/Swiss-Prot database (www.uniprot.org). Signal peptides were predicted using SignalP 4.0 (www.cbs.dtu.dk/services/SignalP) and conserved domains from database models were predicted using external source database SMART (smart.embl-heidelberg.de).

### Quantification and localisation of putative biomineralisation transcripts

Seven putative biomineralisation genes were selected for tissue distribution expression profiling and *in situ* localisation (Supplementary Table S1). Five were selected as they were present in the nacreous shell proteome and two were highly expressed in the previously published mantle transcriptome^12^,^14^. These included a mix of well-characterised biomineralisation candidates such as Pif^16^ and the Tyrosinases^17^,^18^,^19^ (TyrA & TyrB), as well as less well-characterised biomineralisation candidates such as Mytilin^20^ and also a novel nacre shell protein Zinc metallopeptidase and two completely novel genes which had either no annotation (Contig 01043), or only showed sequence similarity to specific domains ([Chitin-binding domain, concavalin-A and Lam-G] (Contig 01311)). Contig numbers refer to previously published transcriptome, assembled contig set available at: http://bit.ly/2cdR1eO and raw reads for assembly are available from the NCBI Short Read Archive Accession PRJNA79569.

#### RNA extraction

Total RNA was extracted from tissues on ice using Tri-Reagent according to the manufacturer’s instructions (Sigma-Aldrich, UK), and purified using RNeasy columns (QIAGEN, UK). All RNA samples were analysed for concentration and quality by spectrophotometry (NanoDrop, ND-1000) and tape station analyses (Agilent 2200 TapeStation). All samples were diluted to 30 ng μL^−1^ total RNA prior to reverse transcription. Following a DNase step, cDNA was synthesised from 1 μg RNA using manufacturer’s protocol (Qiagen, QuantiTect Reverse Transcription Kit). cDNA was stored at −20 °C until further analysis.

#### Gene expression tissue profiling by semi-quantitative PCR

Reproductively mature animals (n = 5, mean shell length = 50 mm +/− 10 mm S.E) were dissected into six different tissues (mantle, siphon, gill, foot, digestive gland and gonad). Gene-specific primers were designed for unique regions of each candidate using Primer 3 software to produce single amplicons with a size of approximately 350–500 bp, annealing temperature of 58–60 °C and GC content between 55–60% (Supplementary Table S1). PCR amplicons were sequenced to confirm identity. cDNA was used as the template in PCR amplification for the seven candidate genes and the *L. elliptica* 18 s gene was used as a positive control and reference housekeeping gene for expression normalisation. Semi quantitative PCR (semi-qPCR) and normalised Integrated Density Value (IDV) calculations were carried out as per ref. 12 with minor temperature specific modifications for each primer set (Supplementary Table S1).

Gene expression data were checked for homogeneity of variance and normality using Levene’s and Kolmogorov-Smirnov’s tests respectively; all data met assumptions of homogeneity of variance and violated the assumption of normality. Data were transformed (Log10[X + 2]) but a normal distribution could not be achieved. Despite non-normal distribution of the transformed data, each tissue was compared using a General Linear Model Analysis of Variance (GLM-ANOVA) followed by post-hoc Tukey test. GLM-ANOVA can handle departures from normality and for added stringency, non-transformed data were also compared using a non-parametric Kruskal-Wallis (K-W) test. Differences between tissues were only considered significantly different if P < 0.05 in both the GLM-ANOVA and the K-W tests.

#### *In situ* hybridisation

Riboprobes (all approximately 1 Kbp) were designed for unique regions of each candidate (Supplementary Table S1) and cloned PCR products were sequenced to confirm identity. Digoxigenin(DIG)-labelled riboprobes were synthesised as previously described in ref. 21. Adult mantle tissues (n = 3) from *L. elliptica* were fixed for 12 h in freshly prepared Davidson fixative (22% formalin, 33% ethyl alcohol, 12% glacial acetic acid and 33% sterile sea water) and transferred to 70% (RT) ethanol for storage. Tissues were embedded in paraffin wax, serially sectioned at 8 μm, mounted onto poly-L-lysine coated slides and dried overnight at 50 °C. Dried tissue sections were rehydrated through a graded ethanol series before being transferred to an Invatis *in situ*-Pro robot for all subsequent treatments as described in ref. 22. In brief, tissue sections were treated with proteinase K (50 μg mL^−1^, 10 min, RT), stopped with 0.2% glycine (5 min, RT), washed with phosphate buffered saline with 0.1% Tween20 (PBTw, 5 min, RT) re-fixed with 4% paraformaldehyde (20 min, RT), incubated with hybridisation buffer (2 h, 55 °C), incubated with specific riboprobe in hybridisation buffer (500 ng μL^−1^, 26 h, 55 °C) and washed with a series of saline-sodium citrate (SSC) buffers (4x, 2x, 1x, 1x with 0.0 1% Tween20, 15 min each, 55 °C). Maleic acid buffer (MAB) was then added to the tissues (10 min, RT), followed by 2% blocking solution (2.5 h, RT) and finally primary anti-DIG antibody conjugated to alkaline phosphatase in 2% blocking solution (1:10,000, 12 h, RT). Unbound antibody was removed with 15 washes in PBTw (20 min, RT) and tissue sections were removed from the robot. For colour development, tissue sections were washed twice with alkaline phosphatase (20 min, RT) before colour detection buffer was added (in the dark, time optimised for each riboprobe to obtain best signal to background ratio, RT). Tissue sections received two final washes with PBTw (5 min, RT) and were post-fixed with 3.7% formamide in phosphate buffered saline (PBS, 2 h, RT) before being dehydrated through a graded ethanol series and mounted with DPX. A list of solutions and full protocol is available in ref. 22.

## Results and Discussion

### Mantle anatomy and cellular ultrastructure characterisation provides map for *in situ* localisation data

**Figure 1:**
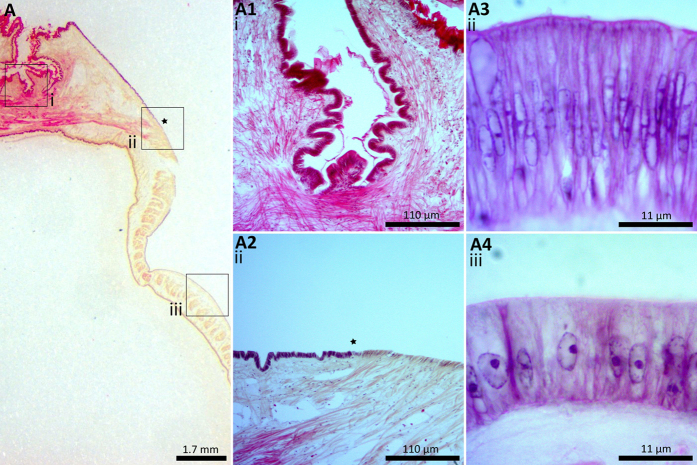
*Laternula elliptica* mantle tissue stained with H&E. (**A**) An overview of tissue anatomy, x0.63 objective, scale bar = 1.7 mm. (**A1**) Fused inner mantle fold and outer mantle folds, x10 objective, scale bar = 110 μm. (**A2**) A region of the right mantle edge, x10 objective, star indicates the start of the pallial attachment, scale bar = 110 μm. (**A3**) A region of mantle edge epithelium, x100 objective, scale bar = 11 μm. (**A4**) = a region of the pallial mantle epithelium, x100 objective, scale bar = 11 μm.

**Figure 2:**
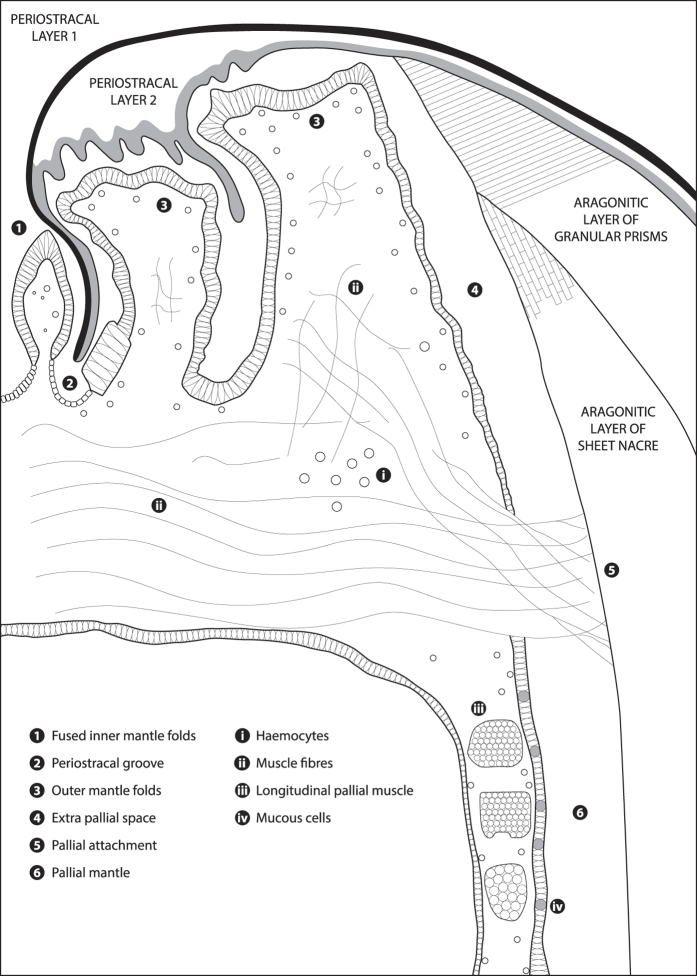
Schematic diagram representing *Laternula elliptica* mantle tissue anatomy, derived from H&E images in Fig. 1 and Supplementary Figure S1. For illustrative purposes only, not to scale.

The anatomy and ultrastructure of the *L. elliptica* mantle tissue was characterised using standard histological staining, LM and TEM techniques to enable the accurate mapping of candidate biomineralisation genes to cell types using *in situ* hybridisation. Knowing precisely where a gene is expressed - at a cellular and subcellular level – aids the interpretation of putative gene function^23^. Based on these histological characterisations (Fig. 1 & Supplementary Figure S1), a schematic illustration of the *L. elliptica* mantle was drawn to aid interpretation of the tissue (Fig. 2). At the mantle edge *L. elliptica* have fused inner mantle folds, a periostracal groove, and what appear to be two outer mantle folds (Figs 1 and 2). The mantle edge is responsible for producing the growing front of the shell, the two periostracal layers and the two shell layers – outer prisms and inner nacre. The enclosed space between the mantle and the shell is the extrapallial space. The mantle attaches to the shell at the pallial line. The mantle edge epithelial cells end and the contractile fibres of the mantle form an attachment in a line around the edge of the shell (pallial line), which continually moves with the growing front of the shell. On the dorsal side of the pallial line, the pallial mantle epithelial cells lay down nacre on the inside of the shell and control the shell thickness^24^. Inside the mantle tissue there are roaming haemocytes (blood cells), contractile fibres and blood sinuses. The haemocytes are part of the mollusc immune system and are also hypothesised to be calcium carbonate chaperones involved in shell repair and growth^25^,^26^.

**Figure 3:**
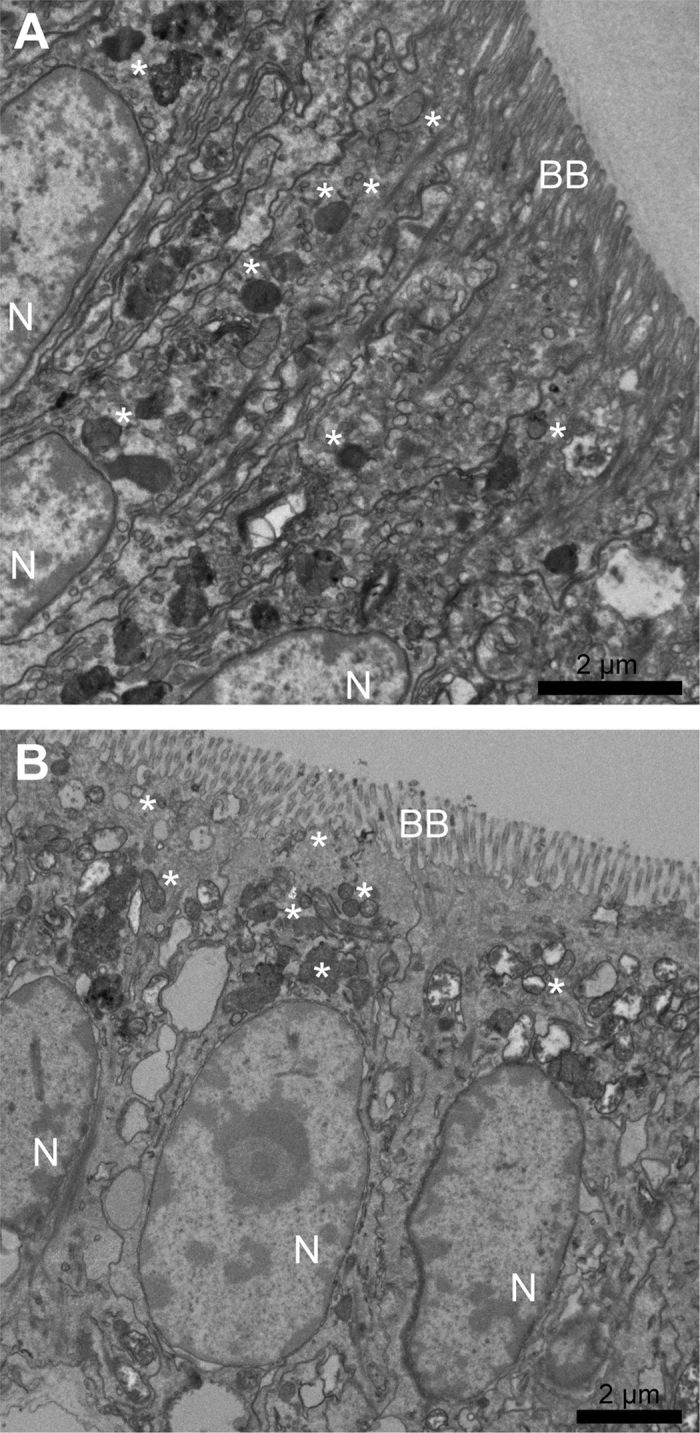
TEM of *Laternula elliptica* mantle epithelial cells. (**A**) Mantle edge epithelium cells, (**B**) Pallial mantle epithelium cells. Asterisk symbols * show examples of secretory vesicles, N denotes nucleus and BB denotes brush border and the shell producing edge. Scale bars = 2 μm.

Similar to other mollusc species^27^, the epithelial cells of the mantle edge are columnar, with an elongate nucleus whereas the epithelial cells of the pallial mantle are more cuboidal with a large basal nucleus (Fig. 3). Both the mantle edge and pallial mantle epithelial cells have electron-dense vesicles. Some of these could contain calcium carbonate and appear to be progressing to the cell apex to be deposited into the extrapallial space, where it is hypothesised that the calcium carbonate moves onto the extracellular protein shell matrix (Fig. 3). Calcium carbonate containing vesicles progressing towards the biomineralisation site have been reported in the mantle epithelial cells of many mollusc species^27^,^28^,^29^, as well as non-mollusc biomineral-producing species^1^,^30^,^31^. An important question regards the form in which calcium carbonate is carried inside the vesicles of biomineral-producing species: is it amorphous, organised, disorganised, solid, liquid, gel or crystalline? Addadi and Weiner recently reviewed biomineral research and discussed the importance of fixation in determining how biominerals are observed^1^, they recommended cryo-fixation as chemical fixation can alter the state of biominerals in cells. For example, if calcium carbonate is present *in vivo* as unstable amorphous calcium carbonate (ACC), fixation can cause it to dissolve or crystallise. Due to logistical constraints, the present study used gluteraldehyde fixations for TEM observations and therefore conclusions concerning the state or species of calcium carbonate inside the vesicles could not be made.

### Shared and unique nacre shell matrix proteins (SMPs)

We identified 37 proteins in the nacreous layer of the *L. elliptica* shell (Table 1). Twenty six proteins were detected with high confidence, either because they were detected independently by the two search engines or because they were identified by more than one peptide. The eleven other proteins were identified with one peptide. Of these identifications, five transcripts were full-length as the conceptually translated contigs had a complete N-terminus and a signal peptide. This suggested that they are secreted by the mantle epithelia through a classic cellular secretion pathway. From the list of identified proteins, most share high sequence similarity with previously described mollusc shell proteins such as, Carbonic anhydrase, Tyrosinase, Shell matrix protein, Mytilin-3, MSI60, Serine protease inhibitor, Chitin-binding protein, Macroglobulin, together with Q-, V-, and S-rich LCD, VWA, Trombospodin, and CBD-2 bearing proteins^32^,^33^,^34^,^35^,^36^,^37^. Additional *de novo* sequencing analyses of MS/MS peptides that were not involved in protein identification showed also the presence of M- and G-rich peptides (Supplementary Table S2). Previous reports have also observed that a M- and G-rich protein, called MRNP34, was present in the shell nacre of the pearl oysters^38^, but to date the function of such domains in SMPs remains enigmatic. Taken together, this nacre SMP list supports the existence of a deeply conserved SMP toolkit of bivalve nacre.

Whilst the majority of the proteins we identified were very similar to previously reported nacre proteins, we found two unique proteins in *L. elliptica* nacre. Firstly, one of the identified proteins contains a Zinc-dependent metalloprotease domain which has not been reported in any shell matrix proteins to date. A second contained a novel Mucin-like protein with remarkable T-rich composition that has also not previously been associated with nacre. Mucins are usually heavily glycosylated and sometimes these sulfated proteins are able to form multimeric insoluble hydrogels through cross-linking. This hydrogel network may form a scaffold within which, nacre can nucleate and develop, due to calcium and carbonate ion saturation^39^. Another Mucin-like protein (Mucoperlin) was previously reported from the shell nacre of the fan mussel *Pinna nobilis*^39^, but it has little sequence similarity with the present *L. elliptica* mucin-like SMP.

### Mantle-specific expression was detected for some - but not all - candidate biomineralisation genes

**Figure 4:**
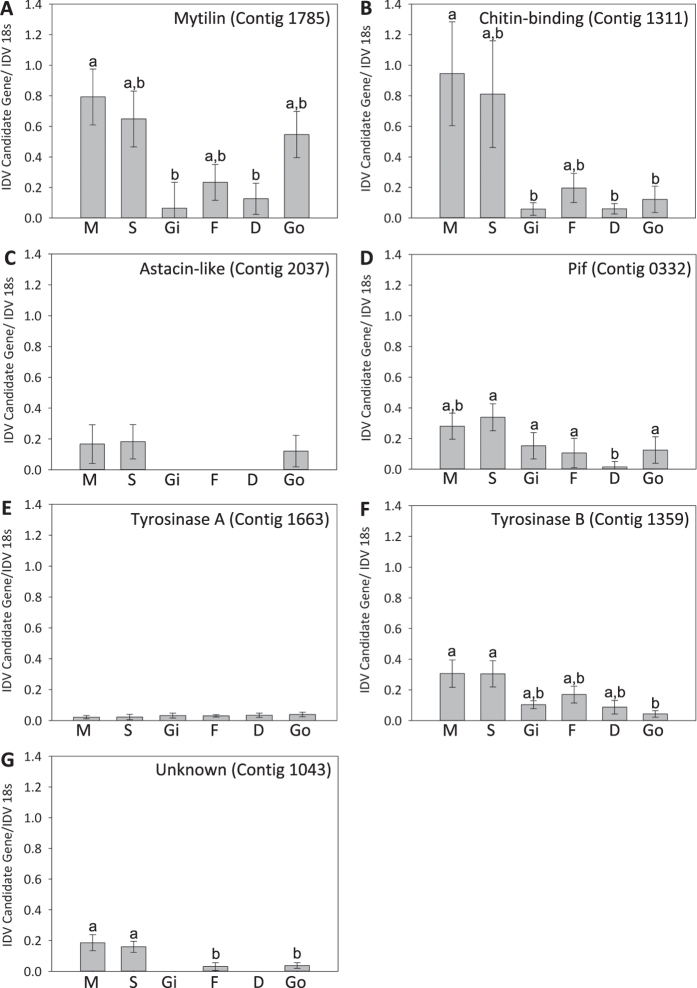
Expression of seven putative biomineralisation genes across six different *Laternula elliptica* tissues as determined by semi-quantitative PCR (mean ± S.E. n = 5). Statistically significant differences indicated by different letters above bars. M = Mantle, S = Siphon, Gi = Gill, F = Foot, D = Digestive Gland, Go = Gonad.

Semi-quantitative PCR demonstrated a mantle/siphon specific expression pattern for four of the seven candidates (Mytilin contig 1785 f = 4.56_29_, P = 0.005, Chitin-binding contig 1311 f = 4.78_29_, P = 0.004, TyrB contig1359 f = 3.76_29_, P = 0.012 and unknown contig 1043 f = 8.75_29_, P = 0.001), supporting their hypothesised role in shell deposition (Fig. 4). Mytilin is an antimicrobial peptide which is produced by haemocytes and its high expression in the mantle is likely due to roaming haemocyctes in the tissue^40^. *L. elliptica* has two copies of Tyrosinase in its genome (TyrA & TyrB) which have been suggested to be the result of a duplication event followed by sub-functionalisation^12^,^41^. Many mollusc species have multiple Tyrosinase paralogues^17^ and another bivalve, *Mytilus edulis*, contains at least two copies which respond differently to acidification stress^42^. Tissue expression profiling revealed *L. elliptica* TyrB had a mantle/siphon-specific expression pattern whereas TyrA showed no difference in expression across tissues and generally had a very low level of expression. Curiously, the proteome of *L. elliptica* shell nacre contained TyrA but not TyrB. Previous work on Tyrosinases in *L. elliptica* showed that the two copies respond differently to shell damage, TyrA is down-regulated and TyrB is up-regulated and has a much higher level of expression overall. In addition previous phylogenetic analysis of amino acid sequences showed that the two Tyrosinases group into distant clades^17^,^20^. The different expression patterns in response to shell damage and their phylogenetic differences, in addition to the tissue distribution expression patterns and shell proteome in the present study, supports the hypothesis that the two copies of *L. elliptica* Tyrosinase are carrying out different functions in the mantle.

The remaining three contigs (Pif, Zn metalloendopeptidase and TyrA) showed a low-level of expression across tissues and a mantle-specific signal was absent. A peak of expression in the mantle is frequently a characteristic of biomineralisation genes^20^,^43^ and it was surprising this pattern was absent for three relatively well-characterised biomineralisation candidates. Antarctic invertebrates such as *L. elliptica* have a low metabolism and grow slowly^44^. One explanation for the low expression of the biomineralisation candidates could be that the animals were not laying down shell at the time they were sampled, or that the rate of shell secretion is unusually slow and therefore difficult to detect at the transcript level (compared to other temperate molluscs which current characterisations are based on). The low-level of expression of these biomineralisation candidates across tissues indicates these genes, and the proteins they code for, could be multi-functional and highlights the need for higher spatial resolution in gene expression data, such as cellular localisation via *in situ* hybridisation.

### Mantle modularity allows for a diverse array of mollusc shells

**Figure 5:**
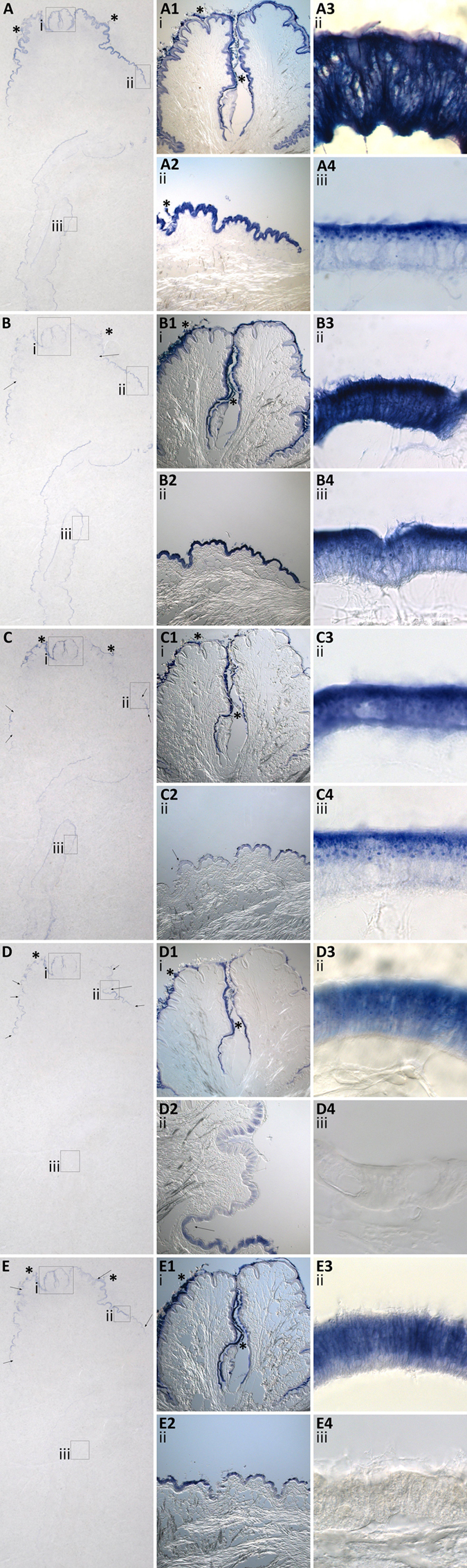
Modular mantle spatial expression patterns of *Laternula elliptica* putative biomineralisation genes. (**A**) An overview of TyrA. (**B**) An overview of Chitin-binding domain. (**C**) An overview of Zn metalloendopeptidase. (**D**) An overview of Pif. (**E**) An overview of Contig 01043 with unknown annotation. Boxes and roman numerals (i, ii, iii) indicate zoomed-in regions, arrows ↓ indicate expression boundaries and asterisk symbols * denote extracellular organic material which is not expression signal. The following corresponding numbers match to the original overview: 1 = fused inner mantle fold and periostracal grooves, x10 objective. 2 = a region of the right outer mantle fold of the mantle edge, x10 objective. 3 = a region of mantle edge outer mantle fold epithelia, x100 objective and 4 = a region of the pallial mantle epithelia, x100 objective. For scale refer to Fig. 1, for schematic representation of the tissue refer to Fig. 2.

The molluscan mantle is anatomically modular in design and can be split into different regions which are thought to be responsible for secreting different layers of the shell (periostracum, prisms or nacre)^27^,^45^,^46^. *In situ* hybridisation of putative biomineralisation transcripts in adult mantle tissue sections revealed that different genes were expressed in different and discrete regions of the calcifying outer epithelium (Fig. 5). Overall five major patterns could be recognised: A) ubiquitous and continuous expression in the outer epithelial cells of the entire mantle edge and pallial mantle (TyrA & TyrB); B) continuous expression in the outer epithelial cells of the entire mantle edge and pallial mantle, which is much weaker in the fused inner mantle fold, and outer mantle folds (Chitin binding domain); C) discrete expression in the outer epithelial cells at the edge of the outer mantle fold next to the pallial attachment and continuous expression in the pallial mantle outer epithelial cells (Mytilin and Zinc metalloendopeptidase); D) discrete expression in the outer epithelial cells on the outer edge of the outer mantle fold which becomes punctate and stops half way along the outer mantle fold with no expression in the pallial mantle outer epithelial cells, the outer epithelial cells of the outer mantle fold next to the pallial attachment, or the outer epithelial cell in the fused inner mantle fold (Pif); E) continuous expression in the outer epithelial cells on the outer edge of the outer mantle fold, with no expression in the pallial mantle outer epithelial cells, the outer epithelial cells of the outer mantle fold next to the pallial attachment, or the outer epithelial cells in the fused inner mantle fold (Contig 01043 with no annotation). The different and discrete gene expression patterns observed here (regardless of their specific details and putative functions) provide further support for the hypothesis that the mollusc mantle is modular in design at the molecular level, as well as the anatomical level^47^. This multi-level modularity, acts as a “blueprint” or framework for molluscan shell production and gives rise to a huge diversity of architecture, microstructure and colour. Despite reports of rapidly evolving and diverse mollusc secretomes at the nucleotide and amino acid sequence level^17^,^47^,^48^, the modularity of the mollusc mantle described here is seemingly a deeply conserved feature present in many different shelled molluscs (fresh water and marine gastropods and bivalves^47^,^49^,^50^,^51^). To what extent such “mantle modules” are truly homologous across molluscan clades, and whether these modules express homologous shell forming genes, remain open questions.

### Functional understanding of Shell Matrix Proteins

Spatial gene expression patterns can be used to infer putative gene function^23^. Mapping gene expression onto different secretory regions of the mantle has the potential to provide a powerful bridge towards a functional understanding of how different genes control the production of specific features of the shell. *In situ* hybridisation revealed that both *L. elliptica* Tyrosinase genes had intense expression in the entire mantle outer epithelium (mantle edge and pallium). Tyrosinase is involved in cross-linking the soluble periostracum precursor (the periostracin) to form an insoluble periostracum^52^ and has previously been localised in the prismatic layer of shell^18^. Both of the *L. elliptica* Tyrosinase paralogues (TyrA & TyrB) were the only candidate genes to be expressed in the fused inner mantle fold, periostracal groove and entire mantle edge, which corroborates with the previously described role of Tyrosinase in the periostracum and prismatic shell matrix. Despite TyrA & B showing different tissue specificities and only TyrA being present in the nacre shell proteome (discussed above), both paralogues have the same spatial expression pattern within the mantle. Curiously, both genes are expressed in the pallial mantle (the region responsible for nacreous shell deposition) yet only TyrA is present in the nacreous shell proteome. TyrB could be involved in nacre formation in the extrapallial space but not become entrapped in the nacre matrix. The weight of evidence strongly suggests TyrA & B are carrying out different functions and there are many different roles TyrB could have in the pallial mantle without being a nacreous shell matrix protein^19^.

The Chitin-binding domain, Mytilin and Zinc metalloendopeptidase genes showed similar expression patterns and were all present in the nacre proteome, with Chitin-binding domain and Mytilin having a mantle-specific tissue expression profile. All three genes were expressed along the entire pallial mantle epithelia and the outer side of the outer mantle fold; however Mytilin and Zn metalloendopeptidase were restricted to a much smaller region, close to the pallial attachment, than the Chitin-binding domain. Mytilin is hypothesised to be multi-functional having roles in the matrix structure of the shell and the immune response as an anti-microbial peptide^40^. It is possible the pallial attachment region is vulnerable to the external environment and hence has a requirement for an increased concentration of anti-microbials or, this region of the shell requires extra reinforcement to accommodate the pallial attachment and hence requires more shell matrix proteins.

Pif and contig 01043 (which has no annotation) showed no expression in the pallial mantle. This is particularly surprising for Pif, as it is classically thought of as a nacre protein^16^,^53^ and indeed it was found in the nacre shell proteome. There are some possible explanations for the lack of expression of Pif in the pallial mantle. Firstly, as previously suggested, *L. elliptica* could be secreting shell much slower than other molluscs, or not at all at the point of sampling. Previous work on Pif expression has shown it to be highly variable^36^. Secondly, Pif’s involvement in nacre deposition could be confined to the growing front of the shell, rather than increasing the thickness of the nacre layers across the whole shell in the pallial mantle. In addition, Pif has been shown to interact with chitin^16^ and it is surprising that its expression only co-localised with the Chitin-binding domain expression at the outer edge of the outer mantle fold. Contig 01043 has no similarity to previously characterised biomineralisation proteins and is not a known shell matrix protein. Since it is expressed in the same discrete set of cells in the mantle edge epithelial as Pif, with which, it shares a very similar tissue expression profile, it could possibly have a similar cellular function, thus ascribing a putative function to a previously “unknown” transcript.

Two subcellular localisation patterns were observed for all candidates: a ubiquitous and strong expression signal in the entire epithelial cell with some vesicle staining (Fig. 5A3,B3,C3,D3 and E3), and expression only in the apical portion of epithelial cells, making vesicle staining easier to visualise (Fig. 5A4,B4 and C4). Cells in the mantle edge epithelia typically showed the ubiquitous subcellular pattern (with the exception of the punctate pattern of Pif), whereas cells in the pallial mantle epithelia showed the apical subcellular pattern. All of the biomineralisation candidates displayed a subcellular expression signal in secretory vesicles. The H&E and TEM mantle characterisations of the *L. elliptica* mantle epithelium (Figs 1 and 3) clearly show large basal nuclei in the epithelial cells with vesicles becoming more concentrated towards the cell apex. Many other molluscs (including various bivalves, gastropods and even shell-producing cephalopods such as *Nautilus pompilius*) show a similar cellular ultrastructure in the mantle epithelium^27^,^28^,^29^. Only one other study has investigated the subcellular localisation of biomineralisation proteins; Fang *et al*.^54^ used antibody protein labelling to observe Calmodulin expression in the nucleus, endoplasmic reticulum and secretory vesicles of *Pinctada fucata* mantle epithelial cells. In the present study seven biomineralisation candidates localised to vesicles, suggesting they may therefore be involved in calcium carbonate transport via vesicle production, chaperoning or secretion.

## Conclusions

Presented here is a multi-disciplinary analysis of biomineralisation in the Antarctic clam *Laternula elliptica*. The mantle tissue anatomy and mantle epithelial cell ultrastructure were described revealing many conserved features with other shell producing molluscs, including secretory vesicles (which could contain calcium carbonate) that progress towards the shell. The proteome of the nacreous shell layer was characterised and 37 shell matrix proteins were identified, many of which corresponded to previously identified mollusc nacre shell matrix proteins, and there were two unique proteins. The expression patterns of seven candidate biomineralisation genes were further investigated to increase understanding of their potential functions. Four genes showed increased expression in the mantle and siphon tissues, and all seven genes had some expression in other tissues, indicating they have multi-functional roles aside from biomineralisation. *In situ* hybridisation of the same transcripts revealed five different and discrete cellular expression patterns which corresponded to different secretory regions of the mantle, providing further evidence that the mollusc mantle is modular at a molecular as well as anatomical level. Subcellular expression patterns suggested that all seven biomineralisation candidates were associated with vesicles, the exact function of which remains unknown, but they may be involved in calcium carbonate transport and secretion. Our analyses suggest that shell matrix proteins not only form the structural matrix required for calcium carbonate crystals to nucleate and grow in a highly organised and regular manner, but may also be important in the vesicular transport of biominerals and immunity.

The *de novo* transcriptome of a non-model organism was first published only eight years ago^55^ and since then, rapidly emerging sequence technologies have been increasingly applied to the field of biomineralisation^14^,^56^,^57^,^58^. A considerable amount of the work on the molecular control of shell production represents the collection and description of much-needed sequence data and initial characterisations of gene and protein expression patterns. Biomineralisation has been revealed to be an incredibly complex process involving the regulation of potentially thousands of genes and tens or hundreds of proteins. Such a complex system is evidently hard to comprehend due to the sheer number of interacting biological variables, each of which being vital for the precise control of shell production. Detangling this immensely complex problem, to understand how molluscs build their shells, and to usefully apply knowledge on the molecular mechanisms underpinning shell production to materials science, aquaculture and ecosystem resilience predictions, requires the continued co-ordination and integration of research efforts. Future work should both continue to thoroughly describe data on genes, proteins, cells and tissues in different species and in addition, molecular biologists should engage with the field of computational modelling, for example gene network models, in order to make sense of the vast amount of data being described.

## Additional Information

**How to cite this article**: Sleight, V. A. *et al*. An Antarctic molluscan biomineralisation tool-kit. *Sci. Rep*. **6**, 36978; doi: 10.1038/srep36978 (2016).

**Publisher’s note:** Springer Nature remains neutral with regard to jurisdictional claims in published maps and institutional affiliations.

## Supplementary Material

Supplementary Information

## Figures and Tables

**Table 1:** Identification of the nacre matrix proteins of *Laternula elliptica* by MS/MS analysis.

Contig reference	Proteomic identification score (nb of unique peptides)		Complete sequence/signalP	Sequence similarity		Protein feature
	Mascot	Peaks		Blast (species)	Smart domain search	
Contig01300	519 (9)	145 (4)	N/-	—	—	Novel uncharacterized protein fragment
Contig03872	496 (10)	147 (7)	Y/Y	—	—	Novel mucin-like protein
Contig02265	464 (7)	130 (3)	N/-	CA (*H. cumingii)*	CA	CA fragment
Contig01663	459 (7)	146 (7)	Y/Y	Tyrosinase 3 (*Crassostrea gigas*)	Tyrosinase	Tyrosinase
Contig01302	335 (6)	137 (4)	N/-	—	—	Novel uncharacterized protein fragment
Contig01311	301 (6)	120 (3)	N/-	Shell matrix protein (*M. californianus*)	2 concavalin-A + LamG-like	SMP-like
Contig17957	252 (4)	112 (4)	N/Y	CA (*C. midas*)	CA	CA fragment
Contig08650	223 (3)	103 (2)	N/-	—	—	LCD protein fragment
Contig01826	203 (4)	100 (3)	N/-	—	—	Novel uncharacterized protein fragment
Contig02037	196 (4)	92 (1)	N/-	Zn metalloendopeptidase (*L. gigantea*)	ZnMC metalloprotease	Zn metalloprotease fragment
Contig01312	194 (3)	104 (3)	N/-	—	—	Novel uncharacterized protein fragment
Contig01301	161 (3)	79 (1)	N/-	—	—	Novel uncharacterized protein fragment
Contig13708	161 (3)	—	N/-	—	—	Novel uncharacterized protein fragment
Contig03798	153 (2)	79 (2)	N/-	—	—	S-rich LCD protein fragment
Contig00830	147 (3)	76 (1)	N/-	Beta-hexosaminidase (*C. gigas*)	Glyco_hydro_20	Chitobiase fragment
Contig02085	135 (2)	76 (1)	N/-	CA	CA	CA fragment
Contig03967	131 (3)	—	N/-	—	—	Q-rich LCD protein fragment
Contig01785	91 (2)	60 (1)	Y/Y	Mytilin-3 (*M. galloprovincialis*)	—	Mytilin-3
Contig02135	82 (2)	—	N/-	—	5 concavalin-A	Novel uncharacterized protein fragment
Contig02086	67 (2)	60 (1)	N/-	—	—	Novel uncharacterized protein fragment
Contig04690	61 (2)	—	N/Y	Insoluble matrix protein (*P. fucata)*	—	MSI60-like fragment
Contig06741	78 (1)	60 (1)	N/-	—	—	Novel uncharacterized protein fragment
Contig05798	70 (1)	54 (1)	N/-	—	—	Novel uncharacterized protein fragment
Contig01291	65 (1)	51 (1)	N/-	Papilin (*H. saltator*)	2 kunitz-like	Serine protease inhibitor fragment
Contig01036	61 (1)	46 (1)	N/-	P-U8 (*P. fucata*)	3 CCP + 3 concavalin-A	Novel uncharacterized protein fragment
Contig02084	—	104 (3)	N/-	CA	CA	CA fragment
Contig02500^#^	62 (1)	—	N/-	Papilin (*S. mimosarum*)	2 kunitz-like	Serine protease inhibitor fragment
Contig05574^#^	62 (1)	—	N/-	Chitin-binding protein (*P. martensii*)	Chitin-binding_3	Chitin binding protein fragment
Contig01703^#^	—	84 (1)	N/-	Alpha-2 macroglobulin (*P. fucata*)	Alpha-2 macroglobulin	Macroglobulin fragment
Contig13709^#^	—	68 (1)	N/-	—	—	Novel uncharacterised protein fragment
Contig01288^#^	—	56 (1)	N/-	—	—	V-rich LCD protein fragment
Contig03070^#^	—	50 (1)	N/-	Alpha-2 macroglobulin (*P. martensii*)	Alpha-2 macroglobulin	Macroglobulin fragment
Contig00332^#^	—	49 (1)	N/-	Uncharacterised protein (*L. gigantea*)	3 trombospondin + 3 concavalin-A + LamG	Novel uncharacterised protein fragment
Contig01639^#^	—	47 (1)	Y/Y	Uncharacterised protein (*C. gigas*)	2 peritrophin-like	Novel uncharacterised protein
Contig00435^#^	—	47 (1)	N/-	Uncharacterised protein (*C. gigas*)	2 VWA	Novel uncharacterised protein fragment
Contig18289^#^	—	44 (1)	N/-	—	2 trombospondin	Novel uncharacterised protein fragment
Contig00456^#^	—	44 (1)	Y/Y	—	—	Q-rich LCD protein

Acc. No. = Accession number; CA = Carbonic anhydrase; CBD = Chitin-binding; CCP = Complement control protein (aka Sushi domain); LamG: Laminin G-like domain; LCD: low complexity domain; VWA = Von Willebrand A; # = identification attempt from only one peptide and with only one of the search engines, presenting only limited confidence.
