# Supplementary material for: An Antarctic molluscan biomineralisation tool-kit
Source: Sci Rep. 2016 Nov 11;6:36978. doi: 10.1038/srep36978 (PMC5105077; doi:10.1038/srep36978)
Supplement: Supplementary Information [file srep36978-s1.pdf]

An Antarctic molluscan biomineralisation tool-kit

Victoria A. Sleight[1,2]*, Benjamin Marie[3], Daniel J. Jackson[4], Elisabeth A. Dyrynda[2], Arul Marie[3] and Melody S. Clark[1]

*Corresponding author. Address: British Antarctic Survey, Natural Environment Research Council, High Cross, Madingley Road, Cambridge, CB3 0ET, UK. Telephone: +44 (0) 1223 221288. Email: viceig15@bas.ac.uk

Supplementary Information:

Supplementary Table S1. Primer and transcript information for candidate putative biomineralisation genes selected for further characterisation. *Laternula elliptica* 18s = housekeeping gene for normalisation. M13 primers used to amplify cloned plasmid insert for riboprobe synthesis.

Transcript information			Primers for Semi-qPCR				Primers for *in situ* localisation			
**Contig I.D**	**Annotation**	**Present in nacre shell layer**	**Forward**	**Reverse**	**Product Size**	**Annealing Temp. (C)**	**Forward**	**Reverse**	**Product Size**	**Annealing Temp. (C)**
N/A	*L. elliptica 18s*	N/A	GGCCGTTCTTA GTTGGTGGA	TCATTAACTGGGG CGATCGG	445	60	N/A	N/A	N/A	N/A
N/A	M13 plasmid region	N/A	N/A	N/A	N/A	N/A	CGCCAGGGTTTTCC CAGTCACGAC	TCACACAGGAAACA GCTATGAC	Insert Specific	58
Contig01785	Mytilin	Yes	CGCCGATATGG ATTTACGTG	GGCGAGACAGAA TTCGGATA	413	60	TTATGCCGCCAATGT TCCCT	AAGGCGAGACAGA ATTCGGA	871	60
Contig 01311	Chitin-binding domain	Yes	CACTGTGCTTC GCTGAACAT	GATCTACGCCCTC GTCAGAG	414	58	AAGCCACTTCCCTCA TTCGG	ACTCTGTTGGGTAT GCAGGC	1096	60
Contig 02037	Zn metallo-endopeptidase	Yes	GAAAACGCCCG ACTTGAATA	TCGGTGTGACATC GTTTGAT	496	58	TGCATGACGGAGGA AAAGCT	TGTTACGCGTGACA GAGGAG	912	60
Contig 00332	Pif	Yes	GGACAACACAT TGGGGTAGG	GGATGTATCGGG GAAAAGGT	350	58	CAATCCGTCCACTTC TGCCT	CCACTGAACTGTTCG ACCGA	1002	60
Contig 01663	Tyrosinase A	Yes	CCATCCGCTATC TGTGGTCC	TCTTTTGCGACCTC AGACCC	399	60	GGTAACCAGGCATG ACGGAA	TACCGCGCCTATCA GAACAC	892	60
Contig 01359	Tyrosinase B	No	CGGCCTCATCG TGATAATCT	GGGAAGATTTTCG AATGCAA	479	58	TGTCACCCAATGTCC TGTCG	ATGACTTCCTGGCCA GCTTC	930	60
Contig 01043	Unknown	No	GGGTCAGCTGG TATCCTTGA	AGCGCTTGCAAAA TTGTCTT	456	60	CCAAGCAGTCCATC GTCCTT	GGTGAATACGGACC CAGGAC	1097	60

Supplementary Table S2. *De novo* sequencing of the nacre tryptic peptides that do not match with *Laternula elliptica* transcriptome according to Mascor search using Peaks software.

	Peptide sequence (>=65%)	ALC (%)	m/z	z	RT	ppm	%age of local confidence (< or = to 50%)
1	**SHPLVASK**	88	419.7683	2	28.434	61.1	66 66 91 94 93 94 95 99
2	**LFALFTAAAK**	86	526.8268	2	31.313	30.9	84 61 96 90 65 88 88 95 95 96
3	**LLADLDLTPR**	86	563.8453	2	27.462	32.6	93 91 93 93 91 89 75 74 91 67
4	**LLEDASTVR**	83	502.2921	2	16.799	34.9	89 84 94 91 89 83 78 80 53
5	**APNEPVLK**	83	434.2661	2	6.916	36.2	97 93 83 87 74 75 80 68
6	**TPPLNLVR**	83	455.2710	2	27.705	-18.5	61 65 87 94 81 95 97 78
7	**GSSSGGSGGSGGGDDNR**	82	705.7964	2	1.666	27.0	36 52 75 95 96 95 95 92 93 96 96 93 88 87 72 73 53
8	**FGTDTAAGLEAAR**	80	640.3312	2	22.502	21.4	70 90 93 94 94 97 95 88 87 60 58 60 52
9	**NGGGGGGFEYGLGGGR**	79	706.3400	2	22.276	26.7	51 65 75 87 83 90 90 76 96 56 75 75 89 78 83 94
10	**AVVLNDDGDASELK**	79	723.3808	2	24.166	29.4	40 45 85 97 83 90 94 92 84 83 82 80 85 58
11	**LVVMMTDSMAK**	79	613.3145	2	23.403	18.7	89 89 95 94 95 92 88 54 52 70 44
12	**LLLTADPGSK**	77	507.8091	2	22.744	27.5	91 90 96 93 82 83 77 51 51 53
13	**FGTDTALGLQK**	77	575.8250	2	23.645	28.2	63 87 85 86 89 93 93 88 56 57 43
14	**EELLSLALVK**	75	557.8592	2	31.677	35.2	59 58 88 94 87 72 67 73 86 61
15	**LASAETPVSK**	75	501.7870	2	4.576	20.1	92 90 90 90 91 80 56 48 59 43
16	**MLDVDGGYGPGAPK**	74	688.8461	2	24.132	24.4	83 81 91 92 86 64 71 92 91 56 54 55 95 25
17	**WNYNLYDYR**	74	653.8143	2	27.289	27.7	0 63 94 80 94 94 95 70 74
18	**ALLSVSSLSVSK**	74	595.8705	2	26.682	29.3	76 77 94 92 89 79 68 64 75 62 64 43
19	**TALALLAVLLK**	74	563.3904	2	40.359	11.6	29 29 48 92 94 92 91 90 89 89 65
20	**TVTYNLYDYR**	73	654.3162	2	29.112	-1.2	74 72 81 87 75 92 69 68 44 67
21	**QLGGAAPLGLPQR**	73	639.3884	2	24.861	29.1	36 74 91 81 80 97 95 92 89 88 43 43 35
22	**YSGGNLQSPLNLVR**	73	759.4036	2	28.261	-4.7	41 41 83 92 79 96 66 94 85 60 48 79 87 63
23	**TPAPVVVSALR**	73	555.3412	2	29.476	6.8	30 32 38 86 96 98 92 82 82 91 66
24	**SPVPSGGLDFSALLGR**	72	786.9266	2	38.480	5.0	26 26 27 67 81 92 89 88 84 69 90 85 86 88 89 61

25	HLGGDLQSPLNLVR	72	759.8990	2	30.204	-31.4	37 37 74 89 92 93 66 94 81 67 58 76 79 59
26	TAADVVFLMDGSR	72	691.3649	2	30.689	32.2	61 61 90 92 95 95 80 94 89 70 36 36 28
27	SSVAAQGLSYVK	71	605.3580	2	22.831	50.7	69 69 69 91 95 56 84 53 46 52 91 72
28	TFDGFDGALDEVR	71	721.3951	2	35.481	85.6	38 38 52 78 58 84 84 86 87 83 83 85 62
29	MAPLQGLMASLNK	71	687.3764	2	33.759	12.8	33 33 79 95 58 91 89 79 78 86 72 73 52
30	LASGSSSETTTSGK	71	656.7969	2	4.627	-27.5	58 55 60 63 37 37 38 77 95 89 89 93 95 99
31	QVALSSVLGPGTADLK	70	778.4589	2	29.355	26.7	26 26 27 77 96 84 83 94 97 85 75 72 65 66 84 57
32	RGSPASVGTTAK	70	566.3206	2	5.874	19.7	50 84 84 93 91 89 78 68 54 51 54 36
33	EGEMSSSGTQGTLR	70	720.3372	2	21.049	16.8	35 35 83 90 97 98 96 92 90 50 50 48 63 46
34	HSGGAAPLGLPGAR	70	630.8752	2	30.585	49.5	34 36 48 63 77 96 96 94 92 92 87 43 40 71
35	ESPPTGAVNLR	69	570.8447	2	22.571	71.2	60 58 77 85 82 55 54 90 71 76 50
36	YRGASMMTTTSGK	69	703.7978	2	23.801	-36.9	27 27 87 89 62 59 45 72 74 81 82 87 94
37	MPTELLVAVHR	68	633.4001	2	35.256	70.8	29 29 79 89 94 96 92 54 53 67 60
38	GQMSSTGGAVQTVPR	68	738.3887	2	18.703	29.2	27 27 44 93 95 92 86 79 81 89 45 44 45 92 72
39	MMNNMGGGGGYAGGAR	67	750.8323	2	20.367	28.9	27 27 39 78 94 94 94 80 90 84 67 58 55 52 58 69
40	RPAALDLAALGLR	66	668.8600	2	40.531	-69.5	26 26 26 75 61 61 64 96 90 88 88 89 62
41	QSYGLGVGGSR	66	540.7914	2	18.101	29.9	27 28 70 97 94 78 73 80 71 51 52
42	MLMSPADTRR	66	589.3068	2	23.924	21.0	74 72 81 85 87 74 70 37 37 37
43	RGHPEPMYGGR	65	628.7682	2	19.357	-52.5	26 25 25 82 84 89 88 91 80 81 36

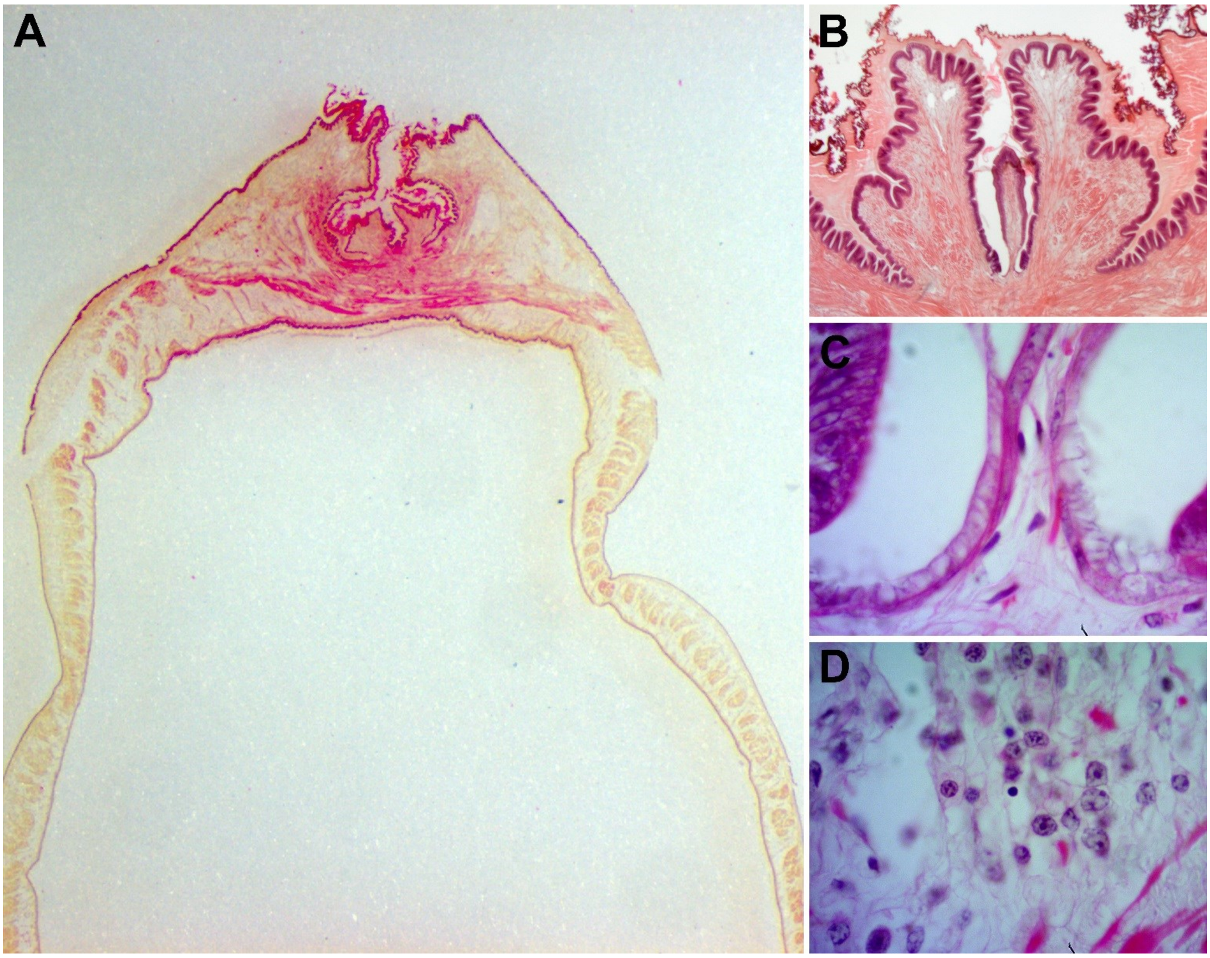

Supplementary Figure S1. Additional *Laternula elliptica* mantle tissue sections stained with H&E to demonstrate variation between individuals and different cell types, refer to Figure 1 for scale. A = an overview of tissue anatomy, x0.63 objective, note the fusion of left and right mantle via the fused inner mantle fold. B = fused inner mantle fold and outer mantle folds, x10 objective, note the two layers of periostracum. C = the periostracal grooves either side of the fused inner mantle fold, x100 objective. D = Roaming haemocyte cells in the mantle tissue, x100 objective.
